# Preoperative oral treatment with cyclooxygenase‐2 inhibitor for cystitis glandularis

**DOI:** 10.1002/iju5.12728

**Published:** 2024-04-08

**Authors:** Masato Yanagi, Norio Motoda, Akifumi Katsu, Hiroyoshi Kono, Ryoji Kimata, Tsutomu Hamasaki, Yukihiro Kondo

**Affiliations:** ^1^ Department of Urology Nippon Medical School Musashikosugi Hospital Kawasaki Kanagawa Japan; ^2^ Department of Pathology Nippon Medical School Musashikosugi Hospital Kawasaki Kanagawa Japan; ^3^ Department of Urology Nippon Medical School Hospital Bunkyo‐ku Tokyo Japan

**Keywords:** bladder tumor, cyclooxygenase‐2 inhibitor, cystitis glandularis, preoperative, ureteral orifice

## Abstract

**Introduction:**

A previous report has shown that cyclooxygenase‐2 inhibitors can prevent the recurrence of cystitis glandularis postoperatively. Herein, we present a case of cystitis glandularis in which the tumor volume was markedly reduced by preoperative oral administration of a cyclooxygenase‐2 inhibitor.

**Case presentation:**

A 45‐year‐old man with voiding difficulty and lower abdominal pain during urination was referred to our hospital. Cystoscopy revealed multiple cystitis glandularis‐like edematous masses on the trigone and the neck of the bladder, completely involving the bilateral ureteral orifices. Cyclooxygenase‐2 inhibitor was orally administered at the patient's request. Six weeks later, the tumor volume was markedly reduced, bilateral ureteral orifices were identified, and the voiding difficulty and pain on urination disappeared. Complete transurethral resection of the residual tumor was performed, and the pathological diagnosis was intestinal‐type cystitis glandularis.

**Conclusion:**

Cyclooxygenase‐2 inhibition can be considered a useful therapeutic strategy for cystitis glandularis.

**Keynote message**

We present a case of CG in which the tumor volume was markedly reduced by preoperative oral administration of a COX‐2 inhibitor. COX‐2 inhibition can be considered a useful therapeutic strategy for CG.

**Abbreviations & Acronyms**
CG: cystitis glandularis
COX‐2: cyclooxygenase‐2
HGUC: high‐grade urothelial carcinoma
MRI: magnetic resonance imaging
RCT: randomized controlled trial
TUR: transurethral resection

## Introduction

CG is a benign, proliferative bladder disease that often presents as multiple masses. The hypothesis that CG is a precancerous lesion is controversial and there is no consensus.^1^, ^2^, ^3^ CG is rare and its etiology and pathogenesis remain unclear. Therefore, few studies have been conducted on treatment methods, and the current standard treatments are TUR and symptomatic treatment.^4^ However, some patients develop resistance to these treatments.^5^

Recent studies have shown that COX‐2 is overexpressed in CG.^3^ COX‐2 is an inducible enzyme present in inflammatory tissues and various tumors.^3^, ^6^, ^7^ COX‐2 functions as an inflammatory mediator that catalyzes the conversion of free essential fatty acids into prostanoids, which are vasodilators. Vasodilation leads to lymphocyte migration, which in turn leads to exacerbation of inflammatory diseases. Based on these facts, a case in which an oral COX‐2 inhibitor was administered for recurrent CG was reported.^8^ The patient was treated with a COX‐2 inhibitor after TUR for recurrent CG, which prevented recurrence.^8^ However, there are no reports of COX‐2 inhibitors as a preoperative treatment for CG. Here, we reported a case in which oral administration of a COX‐2 inhibitor as preoperative treatment markedly reduced the tumor volume in extensive CG.

## Case presentation

**Fig. 1:**
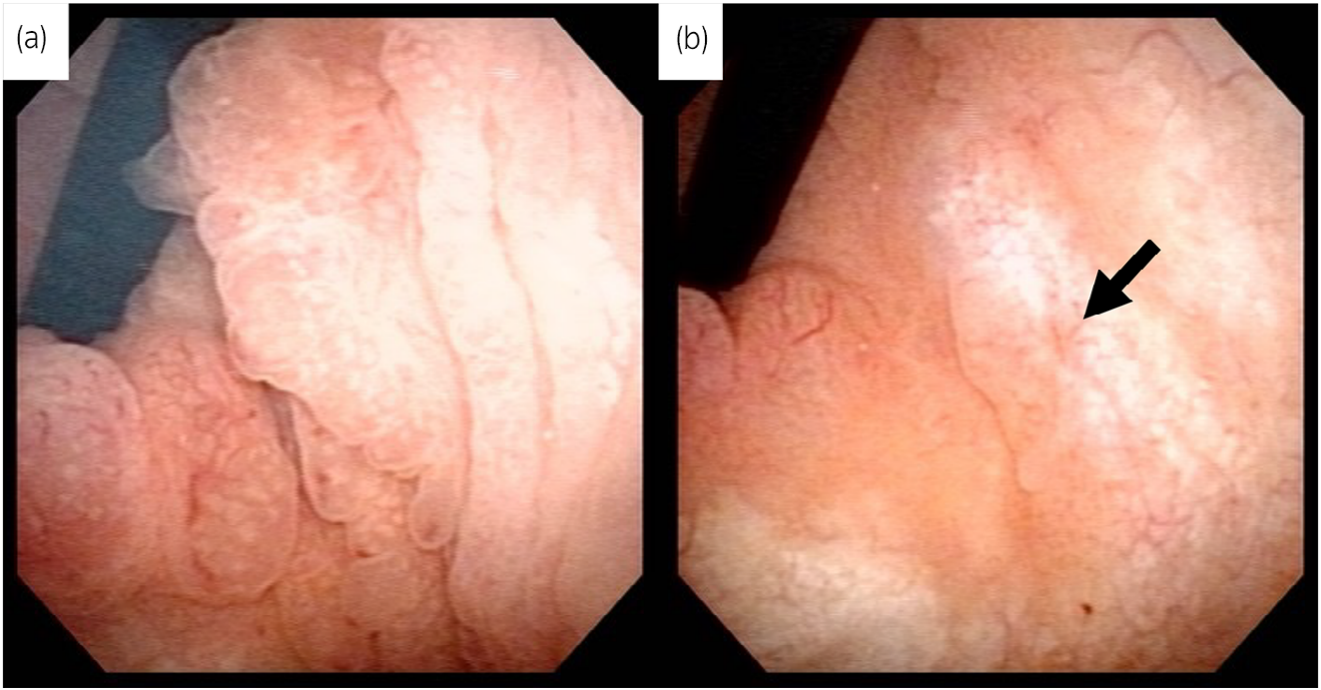
Cystoscopic findings. (a) Cystoscopic findings before oral administration of COX‐2 inhibitor. Cystoscopy revealed multiple extensive CG‐like edematous tumors in the trigone and bladder neck obliterating the bilateral ureteral orifices. (b) Cystoscopic findings 6 weeks after oral administration of COX‐2 inhibitor. Bilateral ureteral orifices can be identified because of tumor shrinkage. Black arrow indicates the left ureteral orifice.

**Fig. 2:**
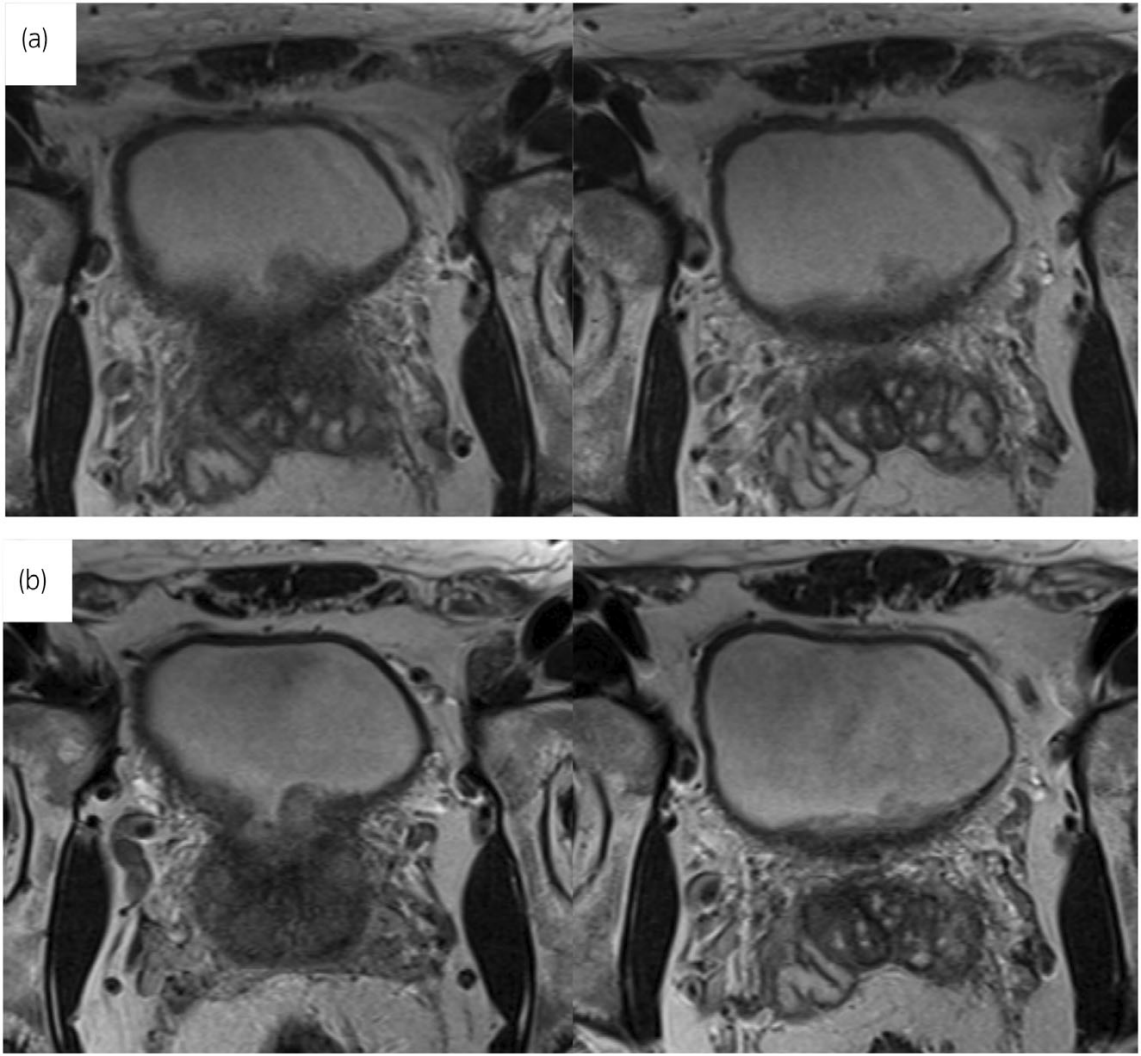
Findings of MRI. (a) Findings of MR before oral administration of COX‐2 inhibitor. T2‐weighted imaging showed a high‐signal thickened bladder mucosa, suggesting proliferative cystitis. (b) Findings of MRI 6 weeks after oral administration of COX‐2 inhibitor. MRI revealed slight tumor shrinkage.

**Fig. 3:**
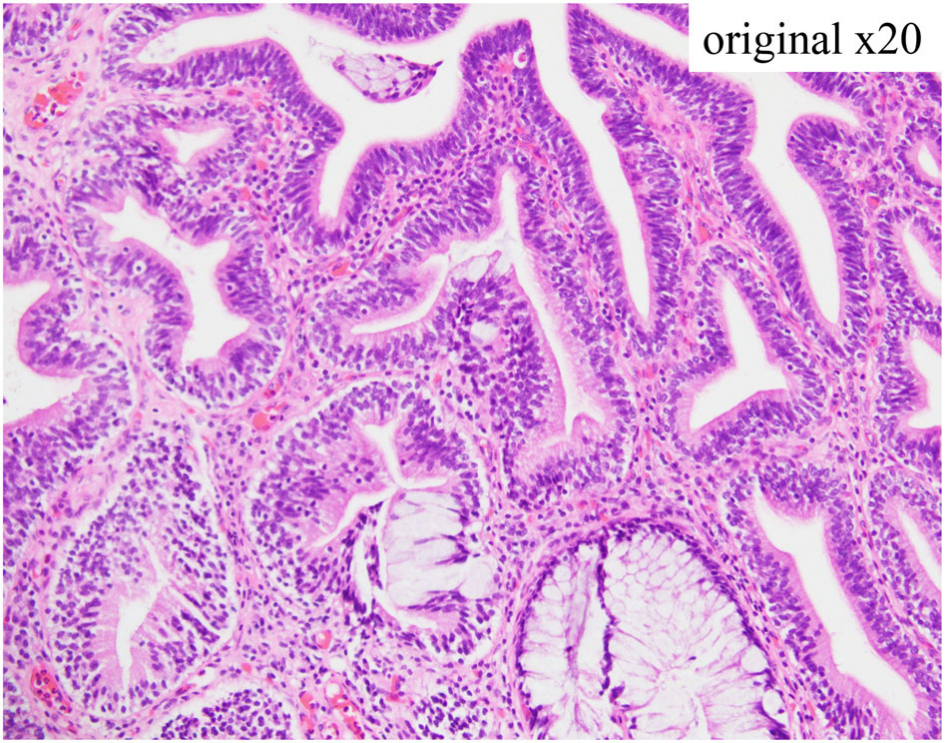
Pathological findings of the tumor. Proliferation of the columnar epithelium with little atypia can be observed, and some colonic‐type glands with goblet cells can be observed in the lower central area of the figure. The stroma is infiltrated by chronic inflammatory cells.

A 45‐year‐old man with mild chronic back pain presented to our department and was thoroughly examined for voiding difficulty and lower abdominal pain during urination. Cystoscopy revealed multiple extensive CG‐like edematous masses in the trigone and the neck of the bladder, involving the bilateral ureteral orifices (Fig. 1a). Morphologically, the lesion was suspected to be CG. T2‐weighted MRI showed a high‐signal thickened bladder mucosa, suggesting proliferative cystitis (Fig. 2a). Ultrasonography revealed no residual urine and computed tomography showed no evidence of hydronephrosis or an upper urinary tract tumor. Urine cytology of The Paris System was negative for HGUC, and the urine sediment showed no hematuria or pyuria. Due to the high possibility of a benign tumor, TUR was scheduled 2 months later. The patient was informed that complete resection of the tumor would be required, however, resection of the bilateral ureteral orifices by TUR, resulting in postoperative complications such as vesicoureteral reflux and ureteral obstruction, and that CG is sometimes resistant to TUR and may recur postoperatively. We also informed him about the case report of postoperative administration of an oral COX‐2 inhibitor that was effective in preventing the recurrence of CG and that the indication for oral COX‐2 inhibitors includes postoperative analgesia after TUR. After this explanation, the patient requested treatment for lower back pain and decided to use an oral COX‐2 inhibitor, which is indicated for chronic lower back pain, with the hope that it would also have an effect on the CG. However, we explained that TUR is necessary for diagnosis because the possibility of malignancy cannot be ruled out. The patient was administered an oral COX‐2 inhibitor (celecoxib, 100 mg twice daily; Astellas Pharma, Tokyo, Japan). Cystoscopy and MRI 6 weeks after the start of oral administration of the COX‐2 inhibitor revealed a significant reduction of the tumor volume, making it possible to identify the bilateral ureteral orifices (Figs 1b,2b). In addition, the lower back pain, voiding difficulty, and lower abdominal pain associated with urination improved. The patient refused TUR 2 weeks later and the COX‐2 inhibitor treatment was continued. Cystoscopy performed 3 months after the start of COX‐2 inhibitor administration revealed no changes in the tumor size compared to 6 weeks before. TUR was performed 4 months after the start of COX‐2 inhibitor administration. The bladder neck and trigone lesions were similar to the preoperative cystoscopy findings. The lesion was completely resected by TUR. During surgery, the bilateral ureteral orifices were easily identified and could be preserved. The histopathological diagnosis was intestinal type CG (Fig. 3). Postoperatively, the administration of a COX‐2 inhibitor was continued for 1 month for postoperative pain and then discontinued. Cystoscopy 3 months after surgery showed no recurrence.

## Discussion

In the present case, 6 weeks after the start of COX‐2 inhibitor administration, significant shrinkage of the lesion was observed and the masses on the bilateral ureteral orifices disappeared. This is the first report of CG shrinkage after oral administration of a COX‐2 inhibitor before TUR.

A COX‐2 inhibitor, celecoxib, has been reported to be effective in the prevention of colorectal adenomas in a RCT.^9^ However, the RCT revealed that long‐term (6 months) administration of celecoxib ≥400 mg/day increased the risk of cardiovascular events as a side effect.^9^ Takizawa *et al*. reported a case of recurrent CG refractory to steroid therapy that was successfully treated with the oral administration of celecoxib after TUR, resulting in the prevention of recurrence.^8^ They administered 100 mg of celecoxib twice daily to avoid cardiovascular events.^8^ The celecoxib dose for back pain was also 100 mg twice daily. Therefore, 100 mg celecoxib twice daily was orally administered in the present case, and significant tumor shrinkage was observed. COX‐2 inhibitors are potentially effective for the treatment of CG because of their anti‐inflammatory effects. It may also be effective in treating bladder irritation symptoms associated with CG. For symptomatic extensive CG, administration of a COX‐2 inhibitor before TUR is a treatment option aimed at symptom palliation and lesion shrinkage. However, a histological diagnosis is necessary because the possibility of malignancy cannot be ruled out, even in cases of CG with extensive tumor‐like lesions. In addition, it is difficult to predict whether COX‐2 inhibitor monotherapy completely eliminates extensive CG. Therefore, TUR is mandatory after the oral administration of a COX‐2 inhibitor. On the other hand, there has been a report of a pediatric patient with CG who showed a significant response to prednisolone for CG refractory to COX‐2 inhibitor treatment.^10^ Based on previous cases and the present case, the administration of a COX‐2 inhibitor and steroid might be considered an adjuvant or neoadjuvant therapy for CG. Considering the various side effects of steroids, COX‐2 inhibitors may be the first‐choice treatment. However, the etiology and pathogenesis of CG, and the mechanisms by which COX‐2 inhibitors and steroids suppress CG remain unclear. Further studies are required to elucidate the etiology and pathogenesis of CG and develop a treatment.

In conclusion, CG can be refractory to standard treatments and COX‐2 inhibition could be a useful therapeutic strategy for CG. Further studies are required to determine the optimal treatment of CG.

## Author contributions

Masato Yanagi: Conceptualization; data curation; writing – original draft. Norio Motoda: Data curation; writing – original draft. Akifumi Katsu: Data curation. Hiroyoshi Kono: Data curation. Ryoji Kimata: Data curation. Tsutomu Hamasaki: Data curation; writing – original draft. Yukihiro Kondo: Writing – review and editing.

## Conflict of interest

The authors declare no conflict of interest.

## Approval of the research protocol by an Institutional Reviewer Board

Not applicable.

## Informed consent

Informed consent for publication has been obtained from the patient described in the reported case.

## Registry and the Registration No. of the study/trial

Not applicable.
